# Potassium-Competitive Acid Blockers as a New Frontier in Acid-Related Disorders and the Role of South Korean Pharmacological Innovation

**DOI:** 10.3390/ph19081168

**Published:** 2026-07-26

**Authors:** Jyotsna S. Ranbhise, Manish Kumar Singh, Hyeong Rok Yun, Sunhee Han, Sung Soo Kim, Insug Kang

**Affiliations:** 1 Department of Biochemistry and Molecular Biology, School of Medicine, Kyung Hee University, Seoul 02447, Republic of Korea; jogm25@khu.ac.kr (J.S.R.); manishbiochem@gmail.com (M.K.S.); foryou018@naver.com (H.R.Y.); sunheehan@khu.ac.kr (S.H.); 2 Biomedical Science Institute, Kyung Hee University, Seoul 02447, Republic of Korea; 3 Department of Biomedical Science, Graduate School, Kyung Hee University, Seoul 02447, Republic of Korea

**Keywords:** potassium-competitive acid blockers, tegoprazan, fexuprazan, South Korea, gastroesophageal reflux disease, *Helicobacter pylori* eradication, *CYP2C19* polymorphism, on-demand therapy, nocturnal acid breakthrough, acid-related disorders

## Abstract

For over three decades, Proton Pump Inhibitors (PPIs) have served as the established primary therapy for acid-suppressive therapy; however, their clinical utility is frequently compromised by their slow onset of action, meal-time dependency, and metabolic variability driven by *CYP2C19* polymorphisms. Potassium-competitive acid blockers (P-CABs) represent a definitive pharmacological shift, offering rapid, reversible, and genotype-independent inhibition of the H^+^/K^+^-ATPase. This review outlines the emergence of South Korea as a global epicenter for P-CAB innovation, predicated upon a strategic partnership between a high domestic burden of *Helicobacter pylori* infection and a rising incidence of refractory gastroesophageal reflux disease (GERD). We analyze the clinical and structural developments of indigenous Korean agents, specifically tegoprazan and fexuprazan, the latter of which features an optimized 9-h elimination half-life engineered to mitigate nocturnal acid breakthrough. A critical evaluation of recent clinical evidence is provided, including the most recent 2024 prospective Phase III data, which demonstrate that 14-day P-CAB-based triple therapy achieves significantly improved eradication rates in high-clarithromycin-resistance environments. Furthermore, we explore the emerging 2025 trend toward personalized, on-demand maintenance therapy and address critical long-term safety considerations, including the duration-dependent risk of metachronous gastric cancer identified in recent multicenter longitudinal studies. Ultimately, the South Korean clinical framework is providing the foundational evidence for a global transition toward P-CAB-centered treatment algorithms, redefining the standards of care in modern gastroenterology.

## 1. Introduction

The management of acid-related gastrointestinal disorders underwent a monumental shift in the late 1980s with the introduction of Proton Pump Inhibitors (PPIs). Following the approval of omeprazole, PPIs quickly supplanted H2-receptor antagonists (H2RAs) as the primary therapeutic mainstay for treating Gastroesophageal Reflux Disease (GERD), Peptic Ulcer Disease (PUD), and *Helicobacter pylori* (*H. pylori*) infections. By irreversibly inhibiting the H^+^/K^+^-ATPase enzyme, PPIs offered a level of acid suppression that was previously unattainable, significantly improving mucosal healing rates and patient quality of life for over three decades [1,2,3]. To date, PPIs remain the foundational cornerstone of acid-related disease management globally, supported by decades of established clinical efficacy, a well-documented long-term safety profile, and significant cost-effectiveness afforded by the widespread availability of generic formulations [4,5].

However, as clinical experience with PPIs grew, so did the recognition of their pharmacological ceiling effect. Despite their efficacy, PPIs possess inherent limitations that prevent optimal therapeutic outcomes in a substantial group of patients. First, PPIs are prodrugs with a short systemic half-life (approximately 1–2 h), requiring enteric coating to survive gastric acidity. Immediate-release formulations utilizing sodium bicarbonate buffers represent a significant pharmacological exception designed for rapid onset. But the majority of conventional delayed-release PPIs are subject to the limitations of enteric protection. This requirement contributes to a delayed onset and often necessitates three to five days of repeated dosing to achieve steady-state acid suppression [6,7]. Furthermore, their strict requirement for administration 30 to 60 min before a meal to coincide [8] with the activation of parietal cell proton pumps creates a significant burden for patient compliance [9].

Beyond these general limitations, the efficacy of PPI therapy is further complicated by regional clinical challenges, particularly within East Asian populations. In South Korea, the high prevalence of *H. pylori* and a rising incidence of GERD have placed a premium on consistent and potent acid suppression. In this context, the metabolic dependency of PPIs on the hepatic cytochrome P450 2C19 (*CYP2C19*) enzyme system becomes a critical hurdle. Because genetic polymorphisms in this system lead to highly variable drug exposure, many Korean patients, specifically rapid metabolizers, experience insufficient acid control [10,11,12]. This unmet medical need in the Korean clinical scenario served as the primary catalyst for the domestic pharmaceutical industry to move beyond PPIs and pioneer the development of the next generation of acid blockers, Potassium-Competitive Acid Blockers (P-CABs).

This review explores the rise in P-CABs, with a specific focus on how South Korean research and development are redefining the global standards for acid-related disease management. By addressing the structural and metabolic failures of previous therapies through a novel mechanism of action, Korean P-CABs have transitioned from experimental alternatives to primary clinical tools. To understand the clinical success of these agents in the Korean market, it is essential first to examine the unique pharmacological properties that distinguish P-CABs from their predecessors at the molecular level.

## 2. Materials and Methods

A comprehensive literature search was conducted across the PubMed, Scopus, Google Scholar, and Web of Science databases to identify relevant clinical and pharmacological studies published through January 2025. The search strategy utilized combinations of the following keywords: ‘potassium-competitive acid blocker’, ‘P-CAB’, ‘tegoprazan’, ‘fexuprazan’, ‘*Helicobacter pylori* eradication’, and ‘gastroesophageal reflux disease’.

To capture the most contemporary clinical advancements and the rapid regulatory transition in South Korea, the inclusion criteria prioritized randomized controlled trials, multicenter Phase III studies, and meta-analyses published between 2020 and 2025. Foundational papers and landmark historical trials published before this period were selectively included to provide necessary context regarding the evolution of acid-suppressive therapy and the established molecular mechanisms of the H^+^/K^+^-ATPase. Only peer-reviewed articles published in English and Korean were included in the primary analysis. Case reports, letters to the editor, and non-peer-reviewed conference abstracts were excluded to maintain the high evidence-based standard of the review.

## 3. Mechanisms and Pharmacokinetics of P-CABs

The clinical utility and rapid market penetration of P-CABs within the South Korean therapeutic scene are fundamentally predicated upon a distinct pharmacological model. The evolution from traditional PPIs to P-CABs represents a critical shift in the methodology of gastric H^+^/K^+^-ATPase inhibition, moving from irreversible covalent modification to a high-affinity, reversible ionic competition.

### 3.1. Molecular Mechanism: Ionic Competition vs. Covalent Binding

The divergence between conventional PPIs and P-CABs is ingrained in their distinct binding kinetics and activation requirements. PPIs are substituted benzimidazoles that act as prodrugs; they are required to undergo an acid-catalyzed cleavage within the parietal cell’s secretory canaliculi to transform into active tetracyclic sulfenamides [13,14]. While this interaction is frequently described as irreversible, it is more accurately characterized as slowly reversible. Research has demonstrated that acid secretion recovery is determined by both the de novo synthesis of new pump proteins and the variable dissociation rates of the PPI from specific cysteine residues [15]. This prolonged covalent interaction nonetheless contributes to the recognized lag phase in therapeutic efficacy [16,17].

**Figure 1:**
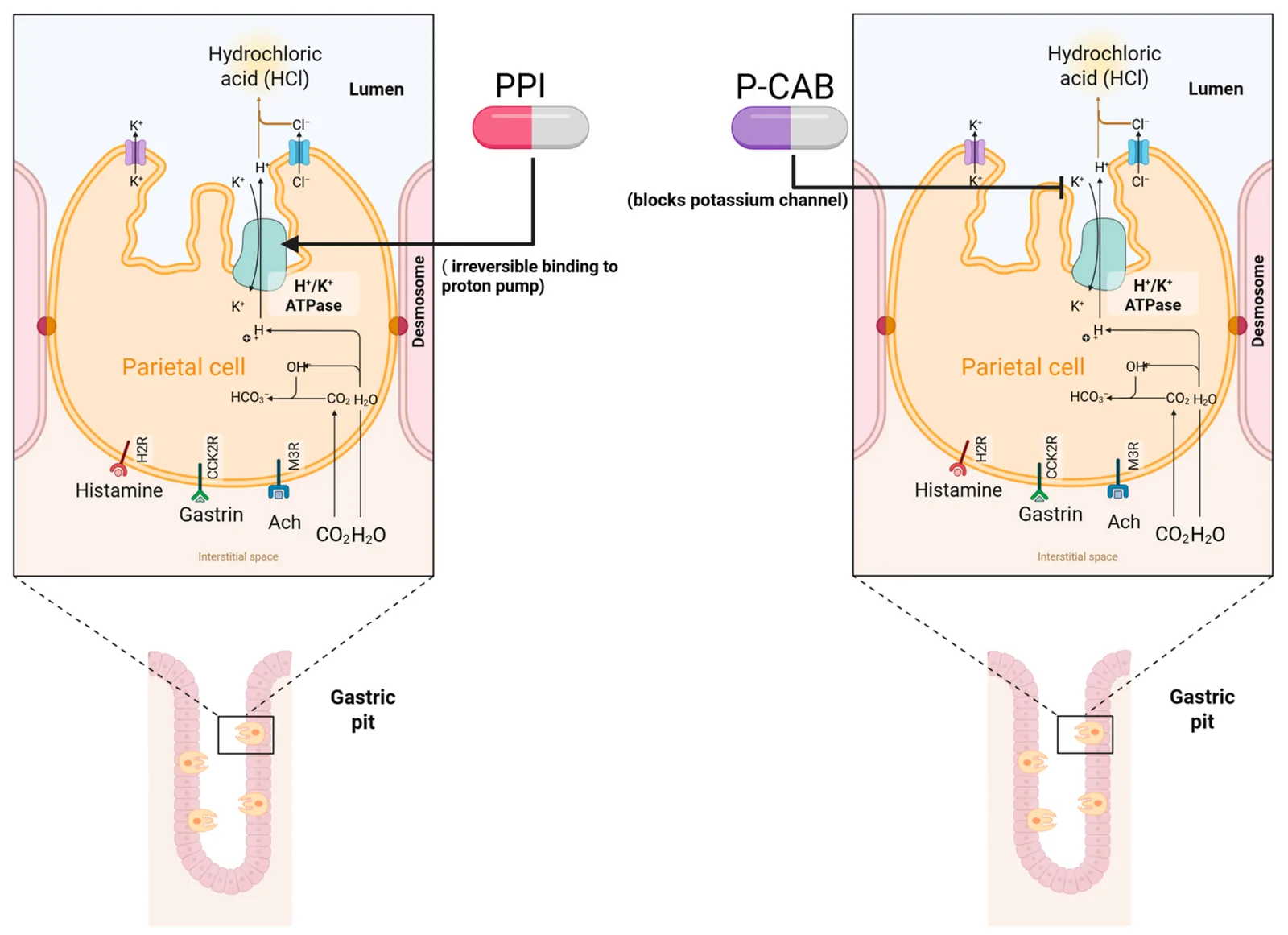
Comparative Molecular and Cellular Mechanisms of Gastric H^+^/K^+^-ATPase. Inhibition by PPIs and P-CABs. The pink capsule (**left**) represents the PPI standard, wherein the prodrug requires acid-catalyzed activation to form an irreversible covalent disulfide bond with the H^+^/K^+^-ATPase, effectively neutralizing actively secreting pumps. In contrast, the purple capsule (**right**) signifies the P-CAB mechanism (e.g., tegoprazan or fexuprazan), which utilizes high-affinity, reversible ionic competition with potassium ions (K^+^) at the luminal exchange site of the ATPase. By physically occluding the K^+^-binding channel, P-CABs inhibit the pump in both active and resting states, facilitating a near-instantaneous onset of acid suppression and ensuring clinical efficacy independent of prandial timing. Created in BioRender. Ranbhise, Jyotsna. (2026) https://BioRender.com/c8llblf (accessed on 25 June 2026).

Conversely, P-CAB agents like tegoprazan and fexuprazan are acid-stable, lipophilic weak bases that bypass the requirement for acid-induced activation [18,19]. These agents function via reversible, non-covalent ionic competition with potassium ions (K^+^) at the pump’s K^+^-binding site [20,21]. By physically occluding the K^+^-exchange channel, P-CABs inhibit the pump in both its active and resting states. This mechanism facilitates a near-immediate inhibition of acid secretion following the initial dose, effectively eliminating the requirement for steady-state accumulation typically seen with PPI therapy [22,23,24]. The divergence in molecular binding kinetics and the resulting impact on the parietal cell’s secretory function are summarized in Figure 1, which provides a comparative structural overview of these inhibitory pathways.

These fundamental differences in binding kinetics transitioning from a slowly reversible covalent modification to competitive ionic antagonism directly dictate the optimized pharmacokinetic and pharmacodynamic profiles of the P-CAB class. While the molecular mechanism provides the foundation for rapid acid suppression, the resulting systemic absorption, distribution, and metabolic stability further differentiate these indigenous agents in clinical practice.

### 3.2. Pharmacokinetic Advantages: Onset, Duration, and Meal Independence

The pharmacokinetic advantage of the P-CAB class over traditional PPIs is primarily due to their high bioavailability and the absence of a required lag phase to achieve therapeutic steady state. Typically, PPIs are susceptible to gastric acid degradation and necessitate enteric coating, which inherently delays systemic absorption. A notable exception includes immediate-release formulations that combine non-enteric-coated PPIs with buffers like sodium bicarbonate to facilitate rapid onset through immediate gastric absorption [25,26]; however, for the majority of conventional PPIs, the requirement for enteric protection results in a delayed *T*_max_. In contrast, P-CABs are acid-stable molecules that facilitate rapid sequestration within the parietal cell canaliculi. Consequently, these agents exhibit significantly accelerated absorption kinetics, with a *T*_max_(time to reach peak plasma concentration) typically occurring within 1.5 to 2.0 h. This rapid systemic absorption facilitates a near-immediate onset of action, characterized by a clinically significant increase in intragastric pH within the first hour of administration [27,28]. From a pharmacodynamic perspective, this rapid systemic residence allows for a maximum inhibitory effect (*I*_max_) on gastric acid secretion following a single oral dose [29,30]. This contrasts sharply with the cumulative dosing required for PPIs, which typically achieve maximal H^+^/K^+^-ATPase inhibition only after three to five consecutive days of administration [31,32,33].

Furthermore, the pharmacodynamic profile of P-CABs is characterized by meal-independent efficacy to address one of the primary compliance barriers of traditional acid suppression [34,35]. Because P-CABs do not require the proton pumps to be in an active, secreting state to facilitate binding, unlike the prodrug mechanism of PPIs, their inhibitory capacity is dissociated from the postprandial state [36]. Pharmacokinetic evaluations have demonstrated that neither the area under the curve (AUC) nor the *C*_max_ (peak concentration) of P-CABs are clinically significantly altered by food intake. This allows for therapeutic flexibility that ensures consistent intragastric pH maintenance regardless of dietary timing, as demonstrated in pharmacodynamic evaluations of healthy volunteers. This is particularly advantageous in maintaining a target pH > 4.0 for extended periods, which is a critical clinical parameter for the healing of erosive esophagitis (EE) and the prevention of mucosal damage [37,38]. In addition to the clinical flexibility afforded by meal-independent pharmacokinetics, the therapeutic predictability of the P-CAB class is further substantiated by its distinct metabolic course. Beyond the external factors of administration timing, the systemic response is further stabilized through an enzymatic pathway that bypasses the significant genetic variability inherent to conventional acid-suppressive regimens.

### 3.3. Predictable Metabolism and the CYP2C19 Advantage

The P-CAB class offers an enhanced inter-individual therapeutic response due to its primary metabolic pathway. The efficacy of conventional PPIs is frequently compromised by the high genetic variability of the *CYP2C19* enzyme system. This variability leads to significant differences in drug exposure between rapid and poor metabolizers. In contrast, P-CABs are predominantly metabolized via the *CYP3A4* pathway [39,40,41]. This metabolic shift results in plasma drug levels that remain more consistent across diverse genetic profiles. This helps mitigate the incidence of refractory symptoms in patients who would otherwise be predisposed to subtherapeutic PPI levels. The relative consistency in the pharmacokinetic and pharmacodynamic (PK/PD) relationship provides a more predictable pharmacological response. Consequently, P-CABs serve as a reliable clinical tool for managing high-prevalence acid-related disorders [42]. These pharmacokinetic attributes provide the foundational rationale for the strategic development of indigenous P-CABs in South Korea. In this region, pharmacological innovation has been systematically integrated into an evolving clinical framework. The distinctive pharmacokinetic and clinical parameters of these agents, including the first-in-class Japanese agent and indigenous Korean optimized molecules, are summarized in Table 1.

## 4. Advancements in P-CAB Development and Clinical Integration in South Korea

The development of the P-CAB class was pioneered by vonoprazan in Japan, which established the clinical feasibility of reversible H^+^/K^+^-ATPase inhibition. Vonoprazan is characterized by a high *pK*_a_ (9.3) and a steady elimination half-life of approximately 7 h. Building upon this precedent, South Korea has emerged as a prominent location for P-CAB innovation, driven by a strategic alignment between clinical necessity and targeted pharmaceutical development. While earlier prototypes demonstrated the feasibility of the class, the South Korean clinical scenario defined by a high seroprevalence of *H. pylori* and a rising incidence of PPI-refractory GERD served as the primary catalyst for the development of second-generation agents [43,44]. The Korean pharmaceutical sector has transitioned the P-CAB class from an experimental pharmacological idea to a clinically dominant standard of care [45,46]. This was achieved through the systematic optimization of binding kinetics and elimination half-lives, resulting in the development of indigenous agents that address specific limitations of first-generation therapy [47]. The clinical exhibition of this industry-wide optimization is best exemplified by the therapeutic route of tegoprazan, the first indigenous Korean P-CAB to achieve comprehensive multi-indication approval and establish a new standard for rapid and consistent acid suppression [48,49].

### 4.1. Tegoprazan: Regulatory Milestones and Indication Breadth

Tegoprazan was approved by the South Korean Ministry of Food and Drug Safety (MFDS) in 2018 as the nation’s 30th novel drug. While first-generation acid blockers focused predominantly on the healing of EE, the development of tegoprazan was specifically brought about to address NERD [50,51]. NERD constitutes the majority of the GERD spectrum in South Korea and is frequently characterized by a suboptimal response to traditional PPI therapy due to the transient nature of acid-reflux events [52].

Tegoprazan is a lipophilic, weak base with a *pK*_a_ of approximately 5.1 [53,54]. This chemical property allows it to highly concentrate within the acidic environment of the secretory canaliculi of the parietal cell without requiring acid-induced transformation into a reactive intermediate [55,56]. The benzimidazole core and chroman moiety allow it to engage in a high-affinity, non-covalent interaction with the gastric H^+^/K^+^-ATPase [57,58]. While PPIs form a covalent disulfide bond with Cys813, tegoprazan functions through ionic competition with potassium ions (K^+^) at the luminal surface of the pump’s α-subunit. It specifically occludes the K^+^binding site, physically preventing the inward transport of K^+^ and the outward transport of H^+^. Because this binding is reversible and avoids covalent modification, tegoprazan facilitates an accelerated onset of acid suppression. This inhibitory effect can be sustained as long as the plasma concentration remains above the threshold for effective H^+^/K^+^ antagonism [59,60,61].

Multicenter, randomized, double-blind Phase III clinical trials conducted across Korean medical institutions demonstrated that a 50 mg dose of tegoprazan maintains an intragastric pH > 4.0 for a mean duration of 18.4 to 20.1 h within a 24-h cycle [62,63]. These values were established in Phase I and II studies involving healthy Korean subjects and symptomatic GERD patients, respectively.

Beyond its application in reflux disorders, tegoprazan achieved a significant regulatory milestone as the first indigenous P-CAB integrated into *H. pylori* eradication protocols in South Korea. The clinical validity of this indication was established through a pivotal multicenter, randomized Phase III trial comparing a 7-day tegoprazan-based triple therapy (50 mg) against a lansoprazole-based triple therapy (30 mg) [64,65]. The trial revealed eradication rates of 62.86% in the Intention-to-Treat (ITT) analysis and 69.33% in the Per-Protocol (PP) analysis for the tegoprazan group. While these absolute rates appear modest, tegoprazan demonstrated clear statistical non-inferiority to the lansoprazole control (60.57% ITT; 67.33% PP), reflecting the challenging reality of primary clarithromycin resistance in South Korea, which has been documented as high as 32.5% to 42.1% in various metropolitan regions [66].

The pharmacological potency of tegoprazan in this context is also driven by its ability to maintain the high intragastric pH required for antibiotic stability. Pharmacodynamic evaluations in healthy Korean subjects have shown that tegoprazan-based regimens maintain an intragastric pH > 6.0 for more than 88% of 24 h, significantly outperforming traditional PPI-based triple therapies [67,68]. This potent acid suppression increases the replicative activity of *H. pylori*, thereby enhancing the bactericidal efficacy of amoxicillin and clarithromycin [69].

Critically, subgroup analyses confirmed that tegoprazan’s efficacy remained consistent regardless of the *CYP2C19* genotype or the minimum inhibitory concentrations of the antibiotics used [70,71]. This emphasizes the agent’s role as a predictable therapeutic option in the Korean clinical setting, where genetic polymorphisms frequently compromise PPI-based outcomes. Furthermore, recent real-world evidence from South Korean multicenter studies suggests that extending the treatment duration to 14 days significantly optimizes outcomes, with tegoprazan-based triple therapy achieving a PP eradication rate of 88.9% [72]. Following the foundational clinical and regulatory milestones established by tegoprazan, the South Korean therapeutic sector pursued further stabilization of acid suppression kinetics, which materialized with the development and subsequent approval of fexuprazan.

### 4.2. Fexuprazan: Pharmacodynamic Optimization and Kinetic Stability

The approval of fexuprazan in 2021 by the South Korean MFDS signified a critical refinement within the P-CAB therapeutic class, specifically targeting the pharmacokinetic limitation of duration of effect prevalent in earlier generations of acid-suppressive agents. While tegoprazan demonstrated the clinical feasibility of rapid, meal-independent acid inhibition, fexuprazan was formulated to maximize systemic residence time and binding stability [73]. It functions as a high-affinity H^+^/K^+^-ATPase inhibitor characterized by an extended half-life (*t*_1/2_ ≈ 9 h) [74,75]. This pharmacokinetic property significantly exceeds the 1.5-h half-life of conventional PPIs and the 4-h half-life typically observed with other P-CABs, facilitating sustained pharmacological activity and consistent intragastric pH maintenance throughout the entire 24-h dosing interval [76,77].

Beyond general systemic kinetics, the extended pharmacodynamic profile of fexuprazan is of huge clinical significance in the management of nocturnal acid breakthrough (NAB), defined as an intragastric pH drop below 4.0 for more than one hour during the night. NAB has been widely discussed as a potential contributor to sleep disruption and esophageal mucosal damage in patients with GERD [78]. However, its direct impact on meaningful clinical outcomes remains a subject of ongoing investigation, as definitive prospective studies linking the prevention of NAB to improved symptomatic relief are currently limited. While pharmacodynamic data demonstrate that fexuprazan maintains a stable, high-affinity interaction with the proton pump throughout the night, further research is warranted to determine if this sustained nocturnal pH maintenance translates into enhanced long-term clinical benefits compared to traditional PPI therapy [79].

The therapeutic efficacy of fexuprazan in this context was specifically validated through a pivotal multicenter, randomized, double-blind Phase III study (*n =* 322) in patients with endoscopically confirmed EE. The trial data revealed cumulative mucosal healing rates at 8 weeks of 88.5% in the Full Analysis Set (FAS) and 97.3% in the Per-Protocol Set (PPS) for the fexuprazan 40 mg cohort. These results established the statistical non-inferiority of fexuprazan to esomeprazole (FAS 89.0%; PPS 97.9%). Notably, in patients stratified with severe Los Angeles classification grade C and D esophagitis, fexuprazan demonstrated robust healing rates and a significantly faster resolution of nighttime heartburn, effectively addressing the therapeutic ceiling effect often encountered with PPI-based regimens [80,81].

Another distinguishing feature of fexuprazan’s pharmacological profile is its linear pharmacokinetic behavior. Unlike the non-linear, saturable kinetics often associated with PPI metabolism (due to *CYP2C19* dependency), fexuprazan’s plasma concentration increases proportionally with the administered dose (up to 320 mg) [82]. This linearity provides clinicians with a highly predictable dose–response relationship, ensuring standardized acid suppression regardless of metabolic polymorphisms within the Korean population. Furthermore, pharmacodynamic evaluations confirmed the absence of a food effect, with fexuprazan maintaining consistent AUC and *C*_max_ levels in both fasted and postprandial states, thereby removing the strict dosing constraints required for optimal PPI efficacy [83,84]. This clinical flexibility, combined with a safety profile, positions fexuprazan as a highly stable agent for the long-term management of chronic acid-related disorders. The structural and clinical refinements achieved with fexuprazan represent the current pharmacological apex of the domestic P-CAB pipeline, providing a critical foundation for examining the broader therapeutic authority and global regulatory course of these indigenous agents.

### 4.3. Clinical Validation and Global Regulatory Integration

The clinical impact of the South Korean P-CAB sector is further evidenced by its role as a clinical incubator for international therapeutic standards. The South Korean healthcare infrastructure, which is characterized by high-volume endoscopic screening and a rigorous regulatory framework, has facilitated extensive post-marketing surveillance and long-term safety evaluations [85,86]. These data have established a strong evidence base regarding the safety and efficacy of P-CABs in populations with high gastric cancer risk and diverse *CYP2C19* genotypes [87].

## 5. Discussion

**Figure 2:**
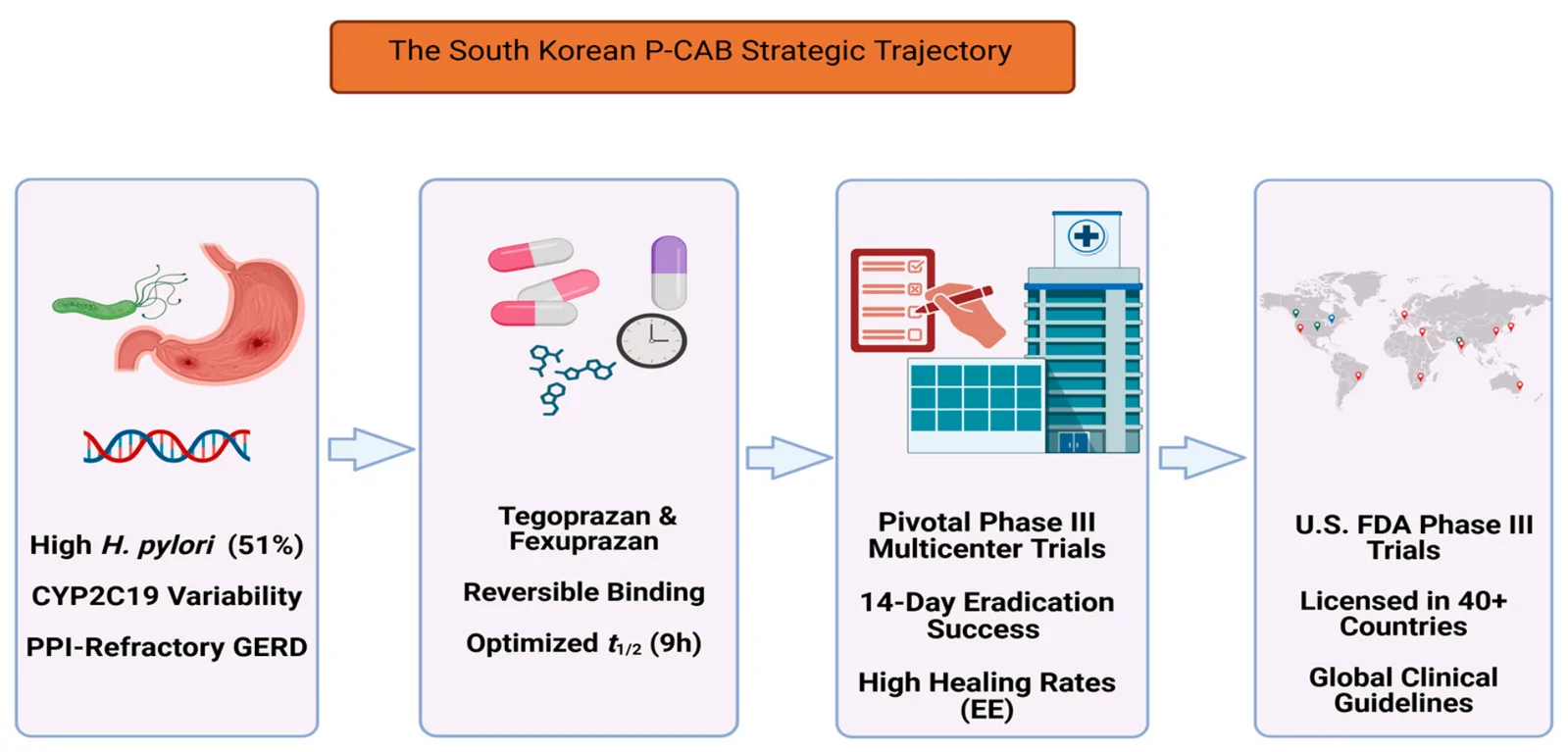
Schematic representation of the South Korean P-CAB clinical and regulatory trajectory. The flowchart delineates the systematic progression from addressing domestic clinical exigencies—specifically the high seroprevalence of *H. pylori* and *CYP2C19*-mediated PPI failure—toward the pharmacological optimization of indigenous agents. This trajectory has culminated in robust clinical validation through domestic Phase III trials and subsequent integration into international regulatory frameworks and global licensing agreements. Created in BioRender. Ranbhise, Jyotsna. (2026) https://BioRender.com/9a7weng (accessed on 21 July 2026).

The pharmacological transition from the slowly reversible covalent inhibition of the H^+^/K^+^-ATPase by traditional PPIs toward the high-affinity, ionic antagonism offered by P-CABs represents a significant therapeutic evolution in gastroenterology [88,89]. This review has outlined how the South Korean therapeutic sector has emerged as a global epicenter for this innovation, predicated upon a strategic synergy between high-burden clinical necessity and targeted structural refinement. The success of the South Korean P-CAB pipeline is intrinsically rooted in its capacity to address the unmet needs of the domestic population. Recent global meta-analyses indicate that the worldwide prevalence of *H. pylori* has decreased from 58.2% in the 1980s to approximately 43.1% in the 2011–2022 period [90,91]. The South Korean clinical scenario is characterized by a persistently high seroprevalence of 51.0% in the adult population. This regional disparity, combined with a rising incidence of PPI-refractory GERD [91], created a demand for agents that supersede the ceiling effect of traditional benzimidazoles. By optimizing the molecular structure of indigenous molecules such as tegoprazan and fexuprazan, Korean researchers have effectively mitigated the metabolic variability associated with *CYP2C19* polymorphisms, which is a primary driver of therapeutic failure in the East Asian rapid metabolizer population [92]. The resulting enhanced pharmacological predictability is essential for maintaining sustained genotype-independent intragastric pH elevation (≥6.0). While healthy volunteer data indicate that this threshold is maintained for the majority of the 24-h period, its clinical importance is most evident in the stability of antibiotics within the gastric lumen of infected patients. This demonstrates a clear optimization, as tegoprazan validated the clinical utility of P-CABs in NERD and eradication therapy [93]. Fexuprazan presented more nuanced pharmacological adaptability, utilizing an extended 9-h elimination half-life to specifically target nocturnal acid breakthrough. The strategic evolution of this developmental pipeline, transitioning from domestic clinical necessity to international regulatory benchmarks, is synthesized in the clinical and regulatory trajectory schematic (Figure 2).

Beyond these pharmacological nuances, the South Korean experience serves as a foundational reference for the global transition toward P-CAB-dominant treatment algorithms. Although the initial 7-day Phase III trials in Korea highlighted the formidable barrier of 21–35% baseline clarithromycin resistance [94]. The subsequent clinical pivot toward 14-day regimens has yielded significantly improved outcomes. A pivotal multicenter, randomized, double-blind Phase III trial (conducted February 2023–April 2024; *n* = 382) demonstrated that 14-day tegoprazan -based triple therapy achieved a modified Intention-to-Treat (mITT) eradication rate of 86.0%, effectively meeting non-inferiority criteria against lansoprazole (78.7%). While these results position P-CAB triple therapy as a robust first-line option, its clinical utility should be evaluated alongside current international guidelines that recommend Bismuth Quadruple Therapy (BQT) or susceptibility-guided treatment in regions with high resistance [95]. BQT remains highly effective. However, it is often limited by a high pill burden and frequent side effects. These issues can negatively impact patient adherence. P-CAB triple therapy offers a more tolerable alternative. It maintains stable pH elevation to optimize antibiotic efficacy without the complexity of BQT. Susceptibility-guided therapy remains the ideal approach for precision management. However, molecular resistance testing is not always accessible in clinical settings. In such cases, P-CABs provide a more reliable empirical platform. Their consistent pharmacological performance, regardless of the patient’s *CYP2C19* genotype, ensures predictable results when resistance data are unavailable [96].

Critically, in the cohort of patients with clarithromycin-resistant infections, tegoprazan exhibited a numerical trend toward high efficacy, with eradication rates reaching 47.8–50.0% compared to 35.5% in the PPI group [97,98]. While this specific subgroup difference did not reach statistical significance, it emphasizes the agent’s capacity to maintain the optimal bactericidal environment (pH ≥ 6.0) required for antibiotic stability, even in high-resistance demographics. Coupled with comparable safety and an absence of drug-related adverse effects, these findings suggest that P-CABs provide a more robust and predictable pharmacology for extended eradication therapy than conventional PPIs [99,100]. The primary clinical trials validating these therapeutic transitions across diverse acid-related pathologies are synthesized in Table 2.

Beyond these regional pharmacological successes, the South Korean clinical experience is serving as a foundational map for the global transition toward P-CAB-centered treatment algorithms. A critical consideration in evaluating South Korean P-CAB research is the extrapolability of these data to Western and other non-Asian populations. Although the initial clinical validation was centered in cohorts with high regional disease prevalence (e.g., *H. pylori* and gastric cancer risk), the core pharmacological advantages of the P-CAB class, specifically their metabolic independence from the *CYP2C19* pathway, suggest robust global generalizability. Because *CYP2C19* polymorphisms exist across all ethnic groups (albeit at varying frequencies), the consistent therapeutic response offered by tegoprazan and fexuprazan addresses a universal clinical challenge [103]. This is further substantiated by recent Western-based Phase III trials, such as the U.S. TRIUMPH trial for tegoprazan, which reported healing rates and safety profiles in American cohorts that were consistent with the foundational data generated in South Korea [104,105]. Consequently, the Korean model of acid suppression appears highly applicable to global treatment models, particularly for patients who remain refractory to traditional PPI-based regimens regardless of their ethnic background.

The global clinical impact of the South Korean P-CAB experience is already evidenced by the evolving site of international gastroenterological guidelines. As the Korean model demonstrates the superiority of 14-day P-CAB regimens in high-resistance environments, international bodies, including the American College of Gastroenterology and the Maastricht VI/Florence consensus [95], are increasingly incorporating P-CABs into their therapeutic algorithms. This signifies a global shift where South Korean clinical data act as a predictive blueprint for Western clinicians [106], particularly in managing the growing population of patients who remain refractory to traditional PPI-based regimens regardless of their ethnic or genetic background.

However, as the clinical focus shifts toward these more potent antagonists, longitudinal research must remain focused on the long-term physiological and oncological implications of profound acid suppression. A significant 2024 multicenter retrospective cohort study from Japan (*n* = 7172) has raised critical concerns regarding the chronic utilization of P-CABs in high-risk populations. The study demonstrated that chronic P-CAB use was independently associated with an increased risk of metachronous gastric cancer (Hazard Ratio [HR] 3.89), a risk notably higher than that observed with conventional PPIs (HR 1.75). Notably, the hazard ratio escalated to 9.36 in patients utilizing therapy for more than three years, suggesting a profound duration-dependent risk path [107]. Importantly, it must be emphasized that these findings represent observational associations rather than definitive evidence of causation. Because these data are derived from retrospective cohorts, the results may be influenced by confounding variables, including the baseline severity of gastric mucosal atrophy, the presence of pre-existing intestinal metaplasia, and the physiological impact of hypergastrinemia. While P-CABs induce a more potent and sustained elevation of serum gastrin than conventional PPIs, theoretically exerting trophic effects on the gastric mucosa. Conclusive longitudinal evidence linking this mechanism to malignant transformation in humans is currently lacking.

While current nationwide post-marketing surveillance data from South Korea indicate a favorable safety profile for short-term and maintenance use, these Japanese findings underscore the necessity for rigorous, long-term monitoring and the emphasis on the shortest effective duration of therapy. In this regard, the potential for personalized, on-demand maintenance regimens may offer a strategy to minimize cumulative systemic exposure while maintaining symptomatic relief. A landmark January 2025 prospective study from South Korea demonstrated that 67.7% of patients preferred on-demand fexuprazan therapy for GERD maintenance, with no significant difference in symptom control compared to continuous daily administration [102]. Such as-needed protocols, enabled by the rapid *T*_max_ of the P-CAB class, represent a significant shift toward patient-centered care and may mitigate the concerns associated with long-term profound acid suppression. Ultimately, this South Korean-led innovation represents a catalyst for a systematic transition in gastrointestinal health management. However, it is essential to note that P-CABs are intended to augment, rather than immediately replace, the existing therapeutic armamentarium. Traditional PPIs continue to play a central role in international clinical guidelines due to their proven reliability in the majority of patient populations. For many patients with mild-to-moderate GERD, the established safety experience and low economic burden of generic PPIs remain highly favorable. Therefore, the adoption of P-CABs is most clinically pertinent for unmet needs such as refractory symptoms, high-resistance *H. pylori* strains, and NAB where the specific pharmacokinetic advantages of the class justify the transition from the established PPI standard [108,109].

## 6. Conclusions

In conclusion, the pharmacological milestones achieved within the South Korean P-CAB sector have fundamentally shifted the therapeutic scenario for acid-related disorders. Through the development and systematic optimization of tegoprazan and fexuprazan, the inherent structural and metabolic constraints of the PPI era, specifically *CYP2C19*-driven variability and delayed onset of action, have been effectively mitigated. By providing rapid, meal-independent, and sustained acid inhibition, these agents offer a clinically advanced therapeutic profile for the management of the most challenging phenotypes of GERD and *H. pylori* infection.

However, it is essential to recognize that traditional PPIs currently remain the primary global standard of care for most indications, supported by an extensive history of clinical safety and cost-effectiveness. P-CABs are positioned to supplement the existing therapeutic tool by addressing specific unmet needs. As this drug class continues its global expansion, the South Korean innovation model serves as a significant catalyst for the evolving area of gastroenterology. This transition from conventional therapy must be accompanied by vigilant longitudinal surveillance to address emerging concerns regarding long-term oncological safety and to refine the role of personalized, on-demand maintenance regimens. Ultimately, the foundational evidence established in South Korea provides a robust basis for a more predictable and patient-centered future in the global management of gastrointestinal health.

## Figures and Tables

**Table 1:** Comparative Pharmacological and Pharmacokinetic Profiles of Conventional PPIs and South Korean P-CABs.

Parameter	Conventional PPIs (e.g., Esomeprazole)	Vonoprazan (Japan)	Tegoprazan (South Korea)	Fexuprazan (South Korea)
Binding Mechanism	Irreversible, Covalent	Reversible, ionic	Reversible, ionic	Reversible, ionic
Acid activation	Required (Prodrug)	Not required	Not required (Active drug)	Not required (Active drug)
Onset of action	Slow (3–5 days for steady state)	Rapid (approx. 1 h)	Rapid (approx. 0.5 h)	Rapid (approx. 1 h)
Half-life	Short (~1.5 h)	~7 h	~4 h	~9 h
Meal dependency	High (Must take 30–60 m pre-meal)	Negligible	Meal independent	Meal independent
CYP2C19 Influence	Significant (Genotype dependent)	Negligible	Negligible	Negligible

**Table 2:** Summary of Pivotal Clinical Trials and Evidence Base for South Korean P-CABs.

Indication	Regimen Duration	Primary Result (Eradication/Healing)	Ref.
*H. pylori* Eradication	7 Days	62.86% (ITT); 69.33% (PP)	[44]
*H. pylori* Eradication	14 Days	86.0% (mITT); 91.0% (PP)	[65]
Erosive Esophagitis	8 Weeks	97.3% (PPS) Healing Rate	[101]
GERD Maintenance	On-Demand	67.7% Patient Preference	[102]

## Data Availability

No new data were created or analyzed in this study. Data sharing is not applicable to this article.
